# Challenges faced by multidisplinary new investigators on addressing grand challenges in global health

**DOI:** 10.1186/1744-8603-10-27

**Published:** 2014-04-15

**Authors:** Carmen Logie, Helen Dimaras, Anny Fortin, Santiago Ramón-García

**Affiliations:** 1 Factor-Inwentash Faculty of Social Work, University of Toronto, 246 Bloor Street West, Toronto, ON M5S 1 V6, Canada; 2 Women’s College Research Institute, University of Toronto, Toronto, ON, Canada; 3 Department of Ophthalmology & Vision Sciences, University of Toronto, Toronto, ON, Canada; 4 Division of Visual Sciences, Toronto Western Research Institute, Toronto, ON, Canada; 5 Division of Hematology/Oncology, SickKids Research Institute, Toronto, ON, Canada; 6 Department of Biochemistry, McGill University, Montreal, Canada; 7 Dafra Pharma Research & Development, Turnhout, Belgium; 8 Department of Microbiology and Immunology, University of British Columbia, Vancouver, British Columbia, Canada; 9 Centre for Tuberculosis Research, Life Sciences Centre, University of British Columbia, Vancouver, British Columbia, Canada; 10 The Hospital for Sick Children, 555 University Ave, Room 7260, Toronto, ON M5G 1X8, Canada; 11 McGill University, McIntyre Medical Sciences Building, 3655 promenade Sir William Osler, Montréal, Québec H3G 1Y6, Canada; 12 Life Sciences Institute, 2350 Health Sciences Mall, Vancouver BC V6T 1Z3, Canada

**Keywords:** Public health, Global health, World health, Interdisciplinary studies, Research, International cooperation, Developing countries, Public health professional

## Abstract

**Background:**

The grand challenges approach aims to spark innovative and transformative strategies to overcome barriers to significant global health issues. Grand Challenges Canada endorses an ‘Integrated Innovation™’ approach that focuses on the intersection of scientific/technological, social and business innovation. In this article we explore themes emerging from a dialogue between the authors, who are multidisciplinary recipients of the ‘Rising Stars in Global Health’ award from Grand Challenges Canada, regarding benefits of engaging in integrated innovation research, and recommendations for how this approach may develop in the future.

**Discussion:**

Our dialogue followed a semi-structured interview format that addressed three topics: 1) reflections on applying an Integrated Innovation™ approach for global health; 2) thoughts on participation in the Grand Challenges 2012 meeting; and 3) authors’ visions of Grand Challenges Canada and the Grand Challenge movement towards 2020. The dialogue was transcribed verbatim and we used thematic analysis techniques to identify, analyze and report themes in the data. Benefits of working using the Grand Challenges approach centered on two themes: a) the potential for scientific breakthrough and b) building interdisciplinary collaborations and a community of scholars. Challenges and opportunities for Grand Challenges in moving forward included: a) capacity building, particularly regarding Integrated Innovation™ and scale-up planning; b) interdisciplinary and international mentorship for new investigators; and c) potential for future commercialization.

**Conclusions:**

Our discussion highlighted that Integrated Innovation™ offers the opportunity to develop new theories, methods and approaches to global health while simultaneously fostering a collaborative spirit grounded in international, interdisciplinary collaborations. However, the arguable over-emphasis on corporatization poses a major challenge for new investigators. We propose a more balanced way forward that can harness technology to foster mentorship across time and space to support the development of such skills and ideas among new investigators.

## Background

“*One bold idea. That*’*s all it takes. Unorthodox thinking is essential to overcoming the most persistent challenges in global health*[1]”.

Research must address problems and develop solutions in order to impact grand challenges in global health. A grand challenge in global health is defined as “a specific critical barrier that if removed would help to solve an important public health problem. The intervention(s) it could lead to might be innovative and, if successfully implemented, will have a high likelihood of impact and feasibility” [2]. This differs from a description of a global health problem (e.g. HIV, malnutrition) in that it involves an innovative and transformative strategy to overcome barriers to significant health issues [2-4].

The grand challenges approach originated in 1900 with the mathematician David Hilbert, who challenged his community to solve 23 major problems remaining in their field. The Bill & Melinda Gates Foundation (BMGF) revived this model and applied it to global health, challenging a global panel of experts to first identify grand challenges in this arena. Twelve grand challenges in 7 broad categories of global health were initially identified [4,5]. The BMGF has been instrumental in spurring high-level technological innovation to tackle these grand challenges with its highly competitive grants program. Since then, additional grand challenges have been identified to address issues in other areas of interest and new organizations have emerged to tackle global health via this approach, including Grand Challenges Canada (GCC). GCC’s funding approach expands beyond the focus on scientific/technological approaches traditionally supported by BMGF, in that they promote Integrated Innovation™ to tackle grand challenges in global health. Integrated Innovation™ is the “the coordinated application of scientific/technological, social and business innovation to develop solutions to complex challenges” [6].

Engaging multidisciplinary voices in the articulation and tackling of grand challenges is key to addressing ethical, social and cultural issues that may arise in global health research [4,5]. There are a wide range of disciplines engaged in health promotion, including immunology, microbiology, clinical sciences, epidemiology, environmental sciences and population/behavioral sciences. Interdisciplinary and international collaboration is transforming the ‘geography of science’ by engaging multiple competencies, theoretical perspectives, and therefore solutions, to address health issues from investigators in diverse locales, disciplines, and sectors [5,7].

While there is great promise for interdisciplinary and innovative approaches to address global health grand challenges, little is known about the experiences of researchers engaging in such endeavors. Especially relevant are the views of new investigators, who are integral to addressing global health challenges [8]. Analyses of social, ethical and cultural issues regarding grand challenges in global health highlighted factors to consider in this area of research. These included community and public engagement strategies, cultural acceptability of approaches, and effective collaboration [9].

In this article we examine the experiences of four new investigators from different disciplines who each received a GCC ‘Rising Stars in Global Health’ award in Round 1 (2011) or Round 2 (2012). During this period a total of 34 such grants were awarded (see http://www.grandchallenges.ca/our-innovators/). The authors met while attending the Grand Challenges meeting co-organized by GCC and BMGF in December 2012, and decided to explore the concepts presented in this debate. The group constitutes a representative sample of the types of innovations that were financed by GCC: projects are linked to health care delivery (CL, HD), diagnostics (HD) and novel anti-infective therapies (SRG, AF) (Table 1). Our projects address myriad health issues across global regions: HIV prevention in post-earthquake Haiti, cancer pathology in Kenya, tuberculosis drug development, and improved drug-delivery against cutaneous leishmaniasis (Table 1).

**Table 1:** Canadian Rising Stars In Global Health Round 1 and 2 projects represented in the analysis

	**Project title**	**Priority area**	**Platform**	**Region**	**Country**	**URL**	**Awards (#)**	**Sample (%)**
***Round 1***							**19**	
CL	Development and evaluation of a tablet-based, communityhealth worker delivered HIV/STI prevention intervention	HIV, Infectious Diseases, Mental Health and Neurodevelopment, Women’s and Children’s Health	Medical/Health Education Programs	Latin America and Caribbean	Haiti	http://www.grandchallenges.ca/grantee-stars/0016-01/		
AF	The use of a permanent make-up (or tattoo) device to target drug delivery against cutaneous leishmaniasis	Infectious Diseases, Leishmaniasis	Medical Devices	Middle East and North Africa	Iran*	http://www.grandchallenges.ca/grantee-stars/0015-01/		
SRG	New therapeutic drug combinations for Tuberculosis treatment	Infectious Diseases, Tuberculosis	Drug development	World-wide	South Africa*	http://www.grandchallenges.ca/grantee-stars/0031-01/		
								16%
***Round 2***							**15**	
HD	Saving Lives: Cancer Pathology in Africa	Cancer	Diagnostics	Sub-Saharan Africa	Kenya	http://www.grandchallenges.ca/grantee-stars/0052-01/		
								7%
						**Summary**:	**34**	**12%**

The authors represent a sample of 3/19 (16%) and 1/15 (7%) Round 1 and Round 2 funded grants, respectively. Projects range in priority area, platform and implementation regions.

*Indicates proposed implementation region.

We found commonality in our commitment to community-based approaches and Integrated Innovation™, and used our heterogeneity as a platform to reflect on our experiences addressing grand challenges in global health. We conducted and digitally recorded a structured dialogue in December 2012 using a semi-structured interview guide to explore three topics: 1) our reflections on applying an Integrated Innovation™ approach in global health research; 2) perceived benefits of our participation in the Grand Challenges 2012 meeting; and 3) our vision for the future of GCC towards 2020. The dialogue was transcribed verbatim and thematic analysis techniques from grounded theory were used to identify, analyze and report themes in the data regarding benefits of the grand challenges approach and recommendations for GCC moving forward [10]. Critical reflexivity in part guided this dialogue; this approach has been used by health practitioners and researchers to interrogate knowledge production processes, and how these processes reproduce or challenge power structures [11,12]. As all of our work aimed to promote health equity among marginalized populations, this was a fitting framework.

## Discussion

**Figure 1:**
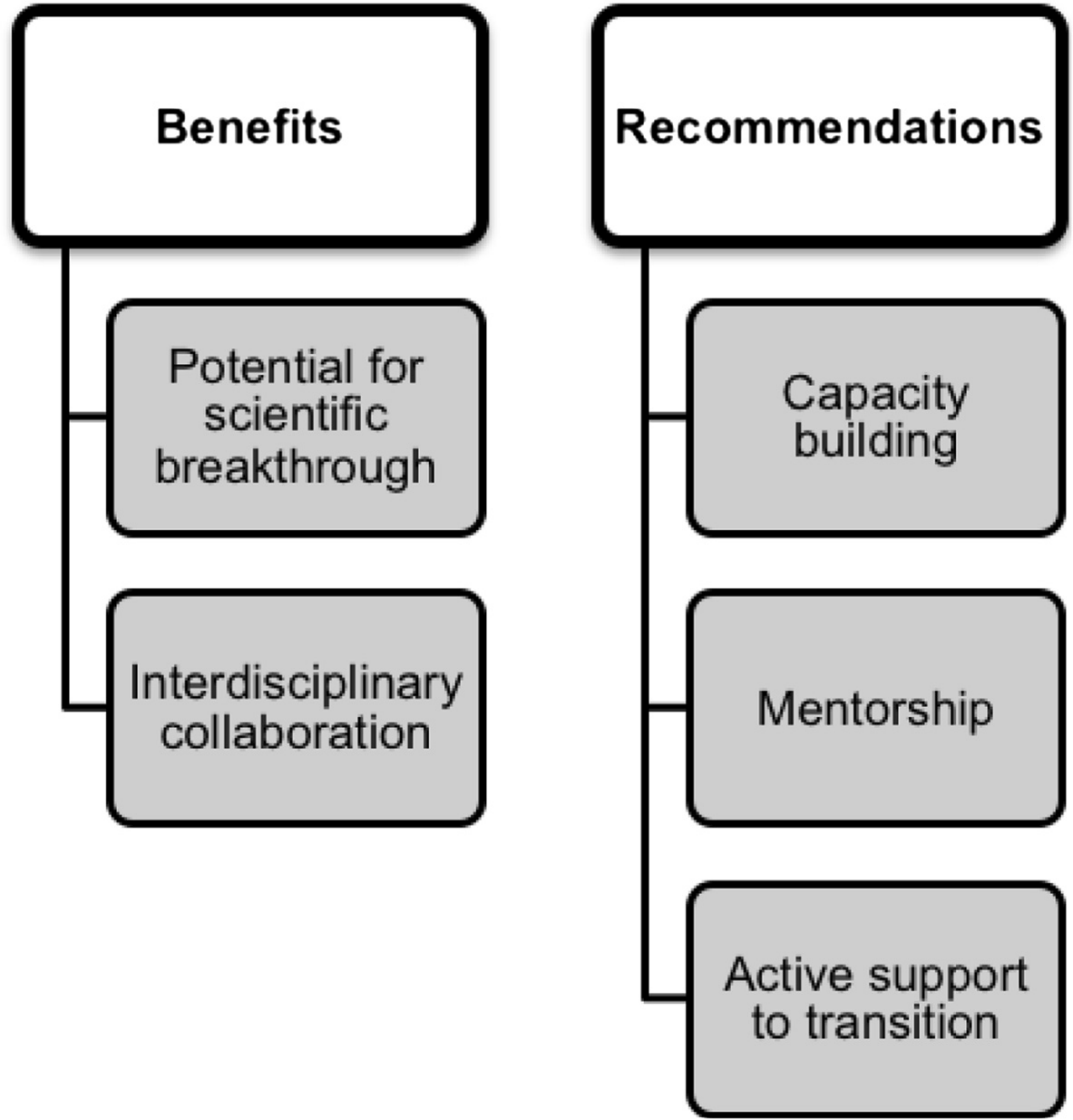
Benefits and recommendations for engagement of new investigators in grand challenges research.

Our discussion, as reported in the following paragraphs, highlighted benefits and challenges of involvement in GCC. A recurring theme in our discussion revealed that while Integrated Innovation™ demonstrates a strong potential for global health impact, an overemphasis on commercialization and corporatization may have the opposite effect. We make recommendations and revisions to be incorporated into the Grand Challenges Canada approach in order to maximize the potential benefit of addressing health challenges on a global scale, particularly when supporting global health research among new investigators (Figure 1).

### Benefits of working within the grand challenges framework

Benefits of working under the Grand Challenges approach centered on two themes: potential for scientific breakthrough and promotion of interdisciplinary collaborations.

#### Potential for scientific breakthrough

The authors discussed the possibilities provided by this funding opportunity that could impact health across sectors as well as build theory. This opportunity was geared at supporting new investigators embarking on innovative proof-of-concept studies for scientific breakthrough. Application of the integrated innovation approach was highlighted as particularly beneficial for education and professional development. One participant described the potential for growth afforded by this funding: “*by awarding this grant*, *GCC is truly challenging us to go beyond our skills and acquire the right knowledge and* (*or*) *collaborators to fit the integrated innovation model*”. Another participant described how the funding could inform work in other contexts: “*if funded*, *my project could really be a blueprint for how community*-*based HIV education is performed in displaced and marginalized populations*…*there are 26 million people living in tents around the world that could benefit from this project*”.

Narratives also underscored the potential of lessons learned to build theory: “*not only are we testing out our individual approaches to specific problems*, *but the success or failure of our projects is really testing out the theory of integrated innovation. That will be a huge addition to global health research*”. Another participant reinforced the importance of learning not only from success but also from failures: “*you don*’*t need to report only the successful discoveries*; *you need to also report the failures*, *as you learn more from the failure than from the success*”. The commitment and persistence to find a solution was articulated: “*the recognition that combating childhood cancer in the developing world is as important as in the developed world is central to my vision*—*I am not going to give up*”.

#### Promotion of interdisciplinary collaboration

One unique benefit of being involved with GCC is the opportunity for interdisciplinary collaboration intrinsic to the Integrated Innovation™ approach: “*You have this mixture of social*, *business and scientific innovators*; *so try to put them together*, *to collaborate and get things done together. This is a very challenging and important thing to do for the future*”. This notion was reinforced by another participant: “*the cool thing about us connecting is that we are out of the silos of our fields of expertise*; *this is really getting transdisciplinary*”. The 2012 Grand Challenge meeting was described as enhancing understanding of business, social and health issues: “*the mixer with the Gates Foundation gave us exposure to perspectives beyond just Grand Challenges Canada*”. The general feeling of partnership was described as something worth fostering: “*Grand Challenges Canada should keep people in a collaborative spirit*; *they should focus on this*”.

Interdisciplinary collaboration was discussed as potentially building partnerships: “*you put a face to the people*; *you start talking to them and seeing things more clearly. You get the feeling that they are real partners*”. This has long-term implications for partnerships to engage in addressing global health issues: “*maybe in 2020 Grand Challenges Canada will have built a network of collaborators between Canadian scientists and scientists in the developed countries to solve the problems of the developing world and to facilitate communication and collaboration*”. The field of Ecohealth (the study of how changes in ecosystems affect human health), is a prime example of how interdisciplinary partnerships can address complex problems related to health by harnessing the collaborative power of multidisciplinary stakeholders: from farmers and community members to healthcare workers, researchers and government.

The importance of developing a community of scholars was also highlighted:

*We need a community of scholars and a community of inquiry*; *a space where anybody who had been a grand challenges star*, *or rising star*, *is part of a larger family*, *a larger community of innovative thinkers. Maybe GCC could have a website or a blog*, *or a meeting for people who are in the same city*, *just so that we can be in touch to keep that community active. That is what I really liked about this meeting*: *we just met each other but it already felt like a community*.

Building a community of grand challenges recipients was articulated as a valuable endeavor: “*these people have got a lot of potential so keeping them in the loop or maybe trying to make a Phase 1 community* [*could be valuable*]”. This community of scholars was described as important for both strengthening society as well as fostering integrated innovation: “*the strength of every society*, *of every institution*, *is their people. So it depends on how much you care for your people. If you get that pool of people moving forward then you are going to be big*, *if you are not able to integrate that*, *you are not going to get integrated collaboration*”.

### Challenges and opportunities

Challenges and opportunities for Grand Challenges Canada to move forward included a focus on capacity building, ongoing mentorship for new investigators, and potential for future commercialization.

#### Capacity building

Participants discussed the need for support beyond financial GCC contribution: “*the social investment of Grand Challenges Canada* [*is important*]. *It is not just their money that we need. For the really impactful innovations we need their time and human resources*”. Commitment to building capacity in new investigators was perceived as critical to long-term success: “*If you are investing time and money in people that you know have talent*, *try and help them move forward. Don*’*t just assume 1 in 10 will be successful*, *but actively support them to increase the ratio to*, *maybe*, *5 in 10 successful stories*”. Capacity building was suggested regarding application of the Integrated Innovation™ approach:

*I really like the idea of integrating the scientific*, *social and business aspects in the development of my idea*, *but at the same time I find it hard because I am not trained in all three. I don*’*t know how often people can manage the three aspects*…*We need to integrate more collaboration in our projects*, *get training and integrate capacity*-*building. To genuinely develop our ideas as integrated innovations*, *we will need the support to do that*.

Scaling up projects and commercialization was identified as another area for capacity building: “*How big the challenge is for us to try to develop integrated innovations given our backgrounds and the type of projects we are working on*! *Left alone we will have to do miracles to get to the commercialization phase*”. The authors felt that the GCC grantee-oriented sessions at the meeting (e.g. workshops covering the power of story telling, networking, grant writing, scale-up planning) were beneficial. Other suggestions for capacity building specifically address integrated innovation and case studies: “*if I had a dream vision for Grand Challenges for 2020 it*’*s actually an online course for all the Phase 1 grantees*, *a lot of information on social innovation*, *on technological innovation*, *on business innovation and successful case studies of how people got funding from Phase 1 to Phase 2*”. Indeed, the authors note that GCC is growing in this area, providing resources for grantees and the public on their website (http://www.grandchallenges.ca/resources/).

#### Mentorship

GCC ‘Rising Stars in Global Health’ awards were originally designed to attract Canadian scientists within ten years of completing a PhD or terminal degree (e.g. M.D.) to validate proof-of-concept proposals that addressed any challenge in global health. These new investigators were required to have a co-investigator in a low-and-middle-income country (LMIC), also within ten years of completing their degree. In a following Phase II application (also called Transition to Scale), these new investigators have the opportunity to apply Integrated Innovation™ to their validated proof-of-concept and scale-up their approach. Currently, the ‘Rising Stars in Global Health’ awards have evolved into ‘Stars in Global Health’ awards now open to new investigators from LMICs, *and* to Canadian investigators at *any* level of experience and expertise. This latter change to the eligibility requirements appears to place new investigators at a disadvantage, as they must compete for Phase II funding against some investigators with presumably greater laboratory capacity, expertise, networks and connections, removing what was perceived as a great opportunity for new investigators to launch their career and develop and test innovative theories and methods. “*This could significantly reduce the chance for new investigators to benefit from the vital Phase I and II opportunities to build research programs*”.

Mentorship was discussed as key to navigating career development, the implementation of Integrated Innovation™ and Phase II scale-up funding applications. Multidisciplinary approaches to mentorship were recommended that included: “*trans*-*disciplinary or multi*-*disciplinary mentorship*, *mentorship from people from the Gates Foundation. Like people who got Phase II and then turned back and mentored Phase I people*, *or people in Phase III can mentor people in Phase II*”. Technological approaches and virtual resources were suggested as an approach to international mentorship: “*Linking* [*grantees*] *to mentors*, *maybe at the Gates Foundation*, *maybe in different countries with previous Phase II winners*, *or a virtual resource that could guide* [*new investigators*]. *In seven years*, *it could be a large enough group to significantly assist future generations* [*of investigators*]”.

Dialogue also focused on the challenges to providing mentorship across such a wide array of research projects: “*Our projects are so individual*, *and therefore genuinely difficult* [*to advise on the transition to Phase II*]”. The need to invest time in mentorship, beyond the context of a meeting, was also highlighted: “*Individual projects are diverse in nature and thus it is difficult to support each one individually at a meeting like this*”.

#### Potential for future commercialization

Integrated Innovation™ promotes a business approach to health projects, in addition to social and scientific components. In theory this approach allows key stakeholders to create partnerships and work towards the resolution of a common problem such a health-related issue. This approach has been successfully implemented in developing countries. However, in the current global neoliberal economy, this approach is often challenging to nearly impossible when the target population of the innovation does not represent a viable market, such as patients suffering neglected diseases or marginalized people living in extreme poverty in low-income countries. Lack of viable markets is a major contributor for diseases being under-researched despite huge medical needs. In addition, many funded projects, including the ones of the authors, have limited or no patent protection and a low-to-null return on financial investment. A participant gave a concrete example: “*We will try to set up a women*’*s cooperative on site but it will be very hard*…*I mean*, *I have to try to create a sustainable business with and for people who make four dollars a month*”. In fact, the GCC Phase I funding strategy could be correlated with a “*survival of the fittest*” approach where a large number of awards are given expecting fewer would progress. Closer interaction and follow up would increase the success ratio. For the Phase II application rounds, GCC is committed to provide 50% matching funding, forcing innovators to establish alliances and partnerships in view of future commercialization. New—as well as more established investigators—are required to seek private sources for this 50% matching funding and are not eligible for federal funding as GCC is partially funded by the Canadian government. Soliciting private funding is an approach that many researchers in academic settings are ill-equipped or trained to do; additionally these investigators often have to navigate goals of internal university fundraising initiatives.

Thus, without new, innovative funding mechanisms in place and implicit GCC support to form those alliances, and alternatives to fund projects serving marginalized populations in low-income countries with few viable markets, many of the Phase I GCC-funded ideas have very little chance to reach commercialization despite achieving proof-of-concept. This impedes contributions to both scientific knowledge and global health promotion. This concept was further highlighted: “*This is not just a question of capacity building. Political authorities and GCC officers would need to facilitate this process by creating new frameworks*, *for example*, *new incentives for large companies to invest in global health*, *or by creating global health foundations*”. Social returns on investments are, however, a tangible outcome for all GCC projects, which may appeal to NGOs or to the so-called “impact investors”, if properly addressed.

In addition, several GCC Phase I projects tackle proof-of-concept studies at the basic science/molecular level. Based on the history of pharmaceutical/biomedicine industry [13], it is unrealistic to assume that these types of projects would reach a good position for commercialization in the Phase II time-frame. An approach to support this type of projects would be to award, in an individual case-by-case basis and after peer-review, Phase Ib grants with clearly established checkpoints in the project development. This strategy, which is already implemented by the BMGF, would help to overcome the large time lag between the proof-of-concept and the scaling and commercialization stage.

## Conclusions and recommendations

In this article we highlight experiences of GCC funding recipients in undertaking research following the principle of Integrated Innovation™, and their recommendations for future GCC initiatives to maximize its impact in solving grand challenges in global health. In particular we focus on the benefits and challenges of engaging in Integrated Innovation™. This includes the potential to realize the aims of the grand challenges approach while building theory and strong evidence of its applicability and utility in addressing global health issues. This approach can inform scientific and technological breakthroughs that address health issues across diverse contexts. Fostering interdisciplinary, international collaborations and a community of scholars dedicated to a global health Integrated Innovation™ approach was identified as a major benefit to the growth and development of new investigators. However, a business focus seriously limits the potential for global health innovations to reach the most marginalized populations in low-income countries who may not present a viable market. If the Integrated Innovation™ focus on financial returns on investments overshadows social and health returns this can reproduce the global health research inequities that neglect diseases of poverty. The widely debated gap in health research, where 10% of global health funding targets diseases that impact 90% of the population, spurred a health equity research agenda [14-16]. Innovation for grand challenges in global health should also interrogate how research approaches can challenge health inequities both within and between countries. Specific recommendations for GCC include:

**Enhanced Support for New Investigators**:

1. Maintain and support the ‘Rising Stars’ award program, whereby a proportion of Phase I and II grants are designated for new investigators. This is an invaluable opportunity to provide new scientists with a platform to test a novel concept and to nurture future generations of global health researchers. This approach is used by other federal funding bodies (e.g. Social Sciences and Humanities Research Council of Canada) that allocates a proportion of Insight Development Grants to new investigators.

2. Integrate a formal mentorship component to their programs, linking new and senior global health researchers, recognizing that the support of new investigators involves more than just financial support. This could take many formats, including meetings in different regions, online platforms, webinars, examples of successful grant applications, etc.

**Promotion of Global Health Scholarship**:

3. Build a Grand Challenges community of scholars for recipients of Phase I/II grants. This could foster collaborations and networking, development of new integrated innovations, and could take many forms, including a website, blog, or meetings for scientists local, national or internationally.

**Support Commercialization and Scale**-**Up**:

4. Develop a strong commercialization support program where grant recipients are linked with potential funders (e.g. NGOs, drug companies, foundations, private investors) either directly or by creating a widely distributed database of funded projects to potential funders. GCC staff members could individually work with grantees to assist them to secure matching funding. For some projects, offering an intermediate ‘Phase Ib’ program could assist investigators in overcoming the large gap between the proof-of-concept and the scale-up/commercialization stage.

5. Value social returns on investment as equivalent to financial returns on investment when working on innovative health projects with marginalized populations in low-income countries.

Building capacity in Integrated Innovation™ and the skills to successfully scale-up the innovations that emerge, in combination with interdisciplinary and international mentorship, will support new investigators in further developing innovative approaches to solve grand challenges in global health. GCC could play a pivotal role at promoting new political frameworks, incentive measures and collective conscious in the business and general community to foster commercialization of innovations addressing the needs of developing communities. Conceptualizing returns on investments as social—not only financial—may be key to promoting global health equity.

## Abbreviations

GCC: Grand Challenges Canada; BMGF: Bill & Melinda Gates Foundation; LMIC: Low-and-Middle-Income Countries.

## Competing interests

The authors declare no competing interests. While all of the authors received Grand Challenges Canada funding, the funders played no role in the conceptualization, writing or publication of this paper.

## Authors’ contributions

Conception and design (CL, HD, AF, SRG); Acquisition, analysis, and interpretation of data (CL, HD, AF, SRG); initial drafting of manuscript (CL); critical revision of manuscript (HD, AF, SRG). All authors read and approved the final manuscript.

## Authors’ information

CL is an Assistant Professor at the Factor-Inwentash Faculty of Social Work at the University of Toronto and an Adjunct Scientist at Women’s College Research Institute. Her GCC-funded project pilot-tested a community-health worker delivered psycho-educational HIV/STI prevention project among internally displaced women in Leogane, Haiti.

HD is an Assistant Professor in the Department of Ophthalmology & Visual Sciences at the University of Toronto, Adjunct Scientist at the SickKids Research Institute and Affiliate Scientist at the Toronto Western Research Institute. Her GCC-funded project focuses on centralization and digitization of cancer pathology services in Kenya.

AF is an Adjunct Member of the Department of Biochemistry at McGill and the Director of Research at Dafra Pharma R&D, a pharmaceutical company dedicated to drug development against neglected diseases. Her GCC-funded project is a proof-of-concept for using a tattooing instrument as a new system to target intra-dermal delivery of drug particles to treat a disease called cutaneous leishmaniasis.

SRG is a Research Associate in the Department of Microbiology & Immunology at The University of British Columbia. His GCC-funded project focuses on developing new therapeutic alternatives for the treatment of tuberculosis repurposing combinations of drugs already approved for other clinical indications.
